# Analysis of Resistance in Magnetic Flux Leakage (MFL) Detectors for Natural Gas Pipelines

**DOI:** 10.3390/s24237563

**Published:** 2024-11-27

**Authors:** Zenggang Zhang, Xiangjun Chen, Chuanmin Tai, Guansan Tian, Guozhao Han

**Affiliations:** School of Thermal Engineering, Shandong Jianzhu University, Jinan 250101, China; zenggang_zhang@sdjzu.edu.cn (Z.Z.); taichuanmin22@sdjzu.edu.cn (C.T.); tgs4170@sdjzu.edu.cn (G.T.); hgz1322@163.com (G.H.)

**Keywords:** magnetic flux leakage, natural gas, frictional resistance, MFL detectors

## Abstract

This study systematically explores the sources and influencing factors of resistance encountered by magnetic flux leakage (MFL) detectors in natural gas pipelines through a theoretical analysis, experimental investigation, and numerical simulation. The research methodology involves the development of a fluid–structure interaction model using ABAQUS 2023 finite element software, complemented by the design and implementation of a pull-testing platform for MFL detectors. This platform simulates detector operation under various interference conditions and quantifies the resulting frictional resistance. The findings reveal that the primary source of frictional resistance is the contact interaction between the MFL detector and the pipeline wall. Key factors influencing the magnitude of this resistance include the detector’s mass, the structural design and materials of the sealing cups and support plates, as well as the surface roughness of the pipeline. Both experimental results and numerical simulations demonstrate a pronounced increase in frictional resistance with heightened interference levels. The theoretical model exhibits strong agreement with experimental data, though deviations are observed under conditions of severe interference. This study provides a detailed understanding of frictional resistance patterns under diverse structural and operational scenarios, offering both theoretical guidance and practical recommendations for the design of low-resistance MFL detectors.

## 1. Introduction

The urban gas pipeline network in China is complex. During actual inspections, not only do pipeline defects and damages affect the operation of MFL detectors but the pipeline’s roughness and diameter variations also influence the detector’s movement and detection efficacy. The driving force and speed of the MFL detector within the pipeline critically affect the accuracy of detection results. If the detector moves too quickly, signal recording and data processing cannot keep pace, resulting in data loss and failure to meet the inspection’s objectives. Conversely, if the detector moves too slowly, inspection time is prolonged, severely impacting efficiency and potentially causing the detector to become lodged in the pipeline, leading to substantial transportation disruptions. The detector’s speed is related to the forces acting upon it, primarily driven by the differential gas pressure at its front and rear ends. During operation, the detector also experiences frictional resistance from contact with the pipeline wall.

Therefore, analyzing the forces acting on the detector, combined with experimental tests to derive its motion patterns and controlling its speed within an optimal range, is essential for ensuring successful inspection operations. Additionally, the movement of the detector within the pipeline can affect the distribution of natural gas. The urban gas network, characterized by numerous facilities, extensive pipelines, and uneven gas consumption among users, further complicates this issue. Studying the impact of the detector’s operation on natural gas supplies is crucial for ensuring normal gas usage by consumers during inspections and for the smooth execution of the inspection process.

Previous research primarily relied on simplified dynamic models of the MFL detector within gas pipelines without considering how variations in the driving components affect frictional resistance in the motion equations. Current numerical methods have mapped the differential gas pressure distribution along the pipeline during stable operation of the detector but have not thoroughly explored the impact of structural design on driving pressure differences. Moreover, these theories lack experimental validation, leading to uncertainties. Additionally, the relationship between the detector’s structural design and the pressure required for movement, as well as the impact of the movement speed on the required pressure differential, has not been addressed.

This study aims to comprehensively explore the sources and influencing factors of resistance encountered by magnetic flux leakage (MFL) detectors in natural gas pipelines through theoretical analysis, experimental research, and numerical simulation. The study includes analyzing the frictional resistance between the detector and the pipeline wall, examining the resistance variation at the cup and plate ends under different interference and thickness conditions, and experimentally validating the accuracy of mathematical models and simulation results.

## 2. Theoretical Basis and Computational Analysis of Resistance in Magnetic Flux Leakage Inspection

### 2.1. MFL Detector

#### 2.1.1. Classification of Pipeline Inspection

The current mainstream international pipeline inspection methods include magnetic flux leakage (MFL) testing, eddy current testing, and ultrasonic testing [1]. Among these, MFL testing is widely used, allowing for pipeline defect detection while maintaining pipeline integrity [2]. MFL testing relies on magnets mounted on the inspection device to magnetize the pipeline’s outer wall. Magnetic field lines near pipeline defects become distorted, and defect identification is achieved by analyzing the magnetic induction data collected by sensors.

Pipeline inspection devices can be categorized into active and passive types based on their driving mechanisms. Active inspection devices are equipped with motor-driven power units, enabling precise speed control during the inspection process. However, their operational range is limited by battery capacity, making them suitable primarily for short-distance pipeline inspections. Passive inspection devices, on the other hand, are typically driven by pressure differentials (either pneumatic or hydraulic) acting on cups within the device. The cups, made from composite materials such as natural rubber and polyurethane, provide elasticity and compressibility. The device moves through the pipeline by maintaining close contact between the cups and the pipeline’s inner wall, ensuring effective sealing and an effective driving force. Throughout the movement, the pressure differential across the cups overcomes frictional resistance to propel the device forward.

#### 2.1.2. Structure of the MFL Detector Device

**Figure 1:**
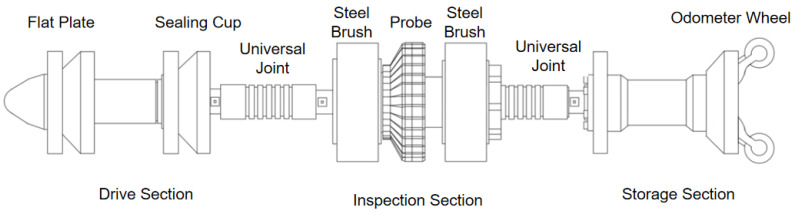
Structure of the MFL detector.

In practical inspections, the MFL detector devices create a sealed environment within the pipeline using cups. Driven by the pressure differential of gas within the pipeline, an MFL inspection device typically comprises a power section, inspection section, and mileage section, as shown in Figure 1.

The drive section provides propulsion for the inspection device, which is driven by a cup mounted on the power section. The cup is designed with a slight interference fit relative to the gas pipeline, allowing it to closely adhere to the inner wall upon entering the pipeline. This interference fit forms a sealed environment where the pressure differential across the cup propels the device. The cup’s elasticity enables the inspection device to smoothly navigate bends and junctions, and it also supports and protects the sensors. The cup’s front end, made of polyurethane flat plates, scrapes away deposits, removes internal water, light oil, methane hydrates, and other corrosive substances as it moves. Inside the cup, a battery provides power for the computer and memory.

The inspection section contains magnets connected to steel brushes. These magnets magnetize both the steel brushes and the pipeline wall, creating a closed magnetic circuit between the permanent magnet, steel brushes, and pipeline wall. When the magnetic field lines encounter a defect, changes in shape reduce permeability and increase magnetic resistance, causing the magnetic lines to deviate and rearrange, generating a magnetic leakage field at the defect location [3]. Probes mounted above the inspection section detect the magnetic leakage signals, allowing for the identification, collection, and differentiation of defect signals on the inner and outer pipeline walls. After detection, specialized data processing software is used to analyze the results and obtain specific defect data.

The storage section, equipped with a computer, performs data analyses, processing, and storage. A mileage wheel is installed at the end to record the movement distance of the inspection device, aiding in defect localization during post-processing. Low-frequency communication is mainly responsible for positioning the device within the pipeline and tracking potential blockages.

The drive section, inspection section, and storage section are connected by universal joints, allowing for a degree of rotational deformation between sections to ensure the device can navigate bends and diameter changes within the pipeline.

#### 2.1.3. Principle of Magnetic Flux Leakage (MFL) Detector for Pipelines

**Figure 2:**
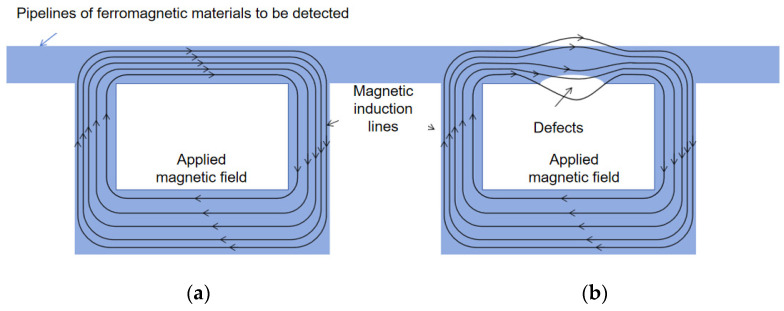
Schematic diagram of the working principle of a magnetic flux leakage (MFL) detector: (**a**) no defects and no magnetic flux leakage; (**b**) with defects and with magnetic flux leakage.

A magnetic flux leakage (MFL) detector for pipelines is based on the principle that ferromagnetic materials exhibit varying levels of magnetic flux leakage in response to defects of different shapes. By correlating the amount of flux leakage with defect geometry, MFL technology can deduce information about pipeline defects. Widely applied in the inspection of ferromagnetic materials, MFL technology leverages the high magnetic permeability of these materials. In a complete, homogenous, and isotropic material, magnetization causes magnetic field lines to concentrate within the material. When a defect is present, the magnetic permeability at that location changes, leading to an increase in magnetic reluctance. This alteration affects the path of the magnetic field lines, with the majority passing through regions of higher magnetic permeability. If the defect is large or the magnetic field strength is significant, the defect cannot accommodate the excess magnetic flux, causing a leakage at the defect site. This leaked flux crosses the defect and re-enters the material, creating a magnetic flux leakage field [4], as shown in Figure 2.

### 2.2. Forms of Resistance Along the Path of the Magnetic Flux Leakage (MFL) Detector

The normal operation of the MFL detector within the pipeline requires overcoming resistance. Analyzing the forms of resistance encountered during its movement is a crucial aspect of understanding its operational dynamics. During operation, the frictional resistance can be categorized into two main types: fluid resistance within the pipeline and frictional resistance due to contact between the MFL detector and the pipeline wall.

While the MFL detector operates, it is entirely surrounded by the medium. Due to the differential speed between the detector and the surrounding medium, relative motion occurs, resulting in viscous resistance from the fluid within the pipeline. In this study, the operating environment of the MFL detector is a natural gas pipeline. The viscous resistance exerted by natural gas on the detector is relatively low and can be approximated as follows:

(1) $$F_{q}=\rho_{q}\cdot S\cdot v_{j}^{2}/2$$

In this equation, $\rho_{q}$ represents fluid density and v_j_ represents the actual operating speed of the MFL detector.

During actual operation, contact between the MFL detector and the pipeline wall results in different friction phenomena, mainly categorized into dry friction and lubricated friction. Dry friction refers to the direct contact and friction between the pipeline and the MFL detector in the absence of lubricants. Lubricated friction occurs when a liquid lubrication layer forms between the cups of the MFL detector and the pipeline during operation, effectively reducing frictional resistance [5]. In practical engineering applications, lubricated friction is typical.

In this study, the model adheres to the applicable conditions for the dry friction approximation, which are outlined as follows:

- Dry Contact Surfaces: The contacting surfaces must remain dry and the gas flowing through the pipeline is a dry gas.
- Distinction Between Static and Dynamic Friction: The dry friction approximation distinguishes between static and dynamic friction. Both types are considered in the experimental research conducted in this study.
- No Wear or Debris Formation: Neither wear nor debris generation occurs during the experimental and simulation processes.
- Steady-State Conditions: Throughout the experiments, other parameters remain relatively constant over time to ensure a stable environment.

Low Relative Motion: Throughout the experimental and simulation process, the relative velocity remains low (approximately 0.5 m/s) where the effects of dry friction are most significant.

Additionally, for feasibility in this study, the friction between the MFL detector and the pipeline wall is simplified as dry friction, with the pipeline medium being gas, the inner wall being kept dry, and a constant friction coefficient being assumed between the detector and the pipeline wall [6].

In the analysis of resistance between the MFL detector and the pipeline, the primary source of resistance is the friction caused by direct contact between the driving cups and the pipeline wall [7]. This friction is influenced by several factors, including the detector’s mass, the structure and material of the driving cups, their deformation, and the roughness of the pipeline.

The mass of the MFL detector significantly affects its operational dynamics. In small-diameter pipeline inspections, frequent start–stop movements of the detector are common. When the MFL detector is stationary, its maximum static friction is related to its mass and is proportional to the normal force it experiences. As the detector’s mass increases, the normal force and maximum static friction also increase, making it more difficult for the detector to start moving. The MFL detector undergoes acceleration and deceleration in the pipeline, with its movement also being influenced by its mass. A larger mass means greater inertia, causing the detector to respond more slowly to external disturbances and making changes in its motion state or direction more challenging.

This study uses the Xionggu Oil and Gas Technology MI Series Full-Speed Ultra-High-Definition Magnetic Flux Leakage Detector, with its parameters being shown Table 1, It originated from Xiong Gu Oil & Gas Technology Co., Ltd. in Chengdu, Sichuan, China.

**Figure 3:**
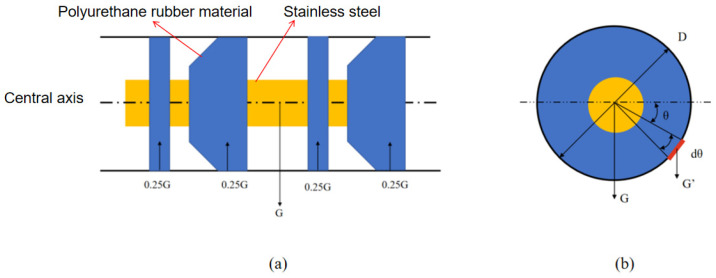
Schematic diagram of the gravitational dynamics of the MFL detector: (**a**) Section View. (**b**) Plan View.

As shown in Figure 3, the MFL detector makes close contact with the steel pipeline’s inner wall via the straight plate and cups. The weight of the detector mainly acts on the lower parts of the plate and cups, generating compressive force through material deformation. Assume the MFL detector has *n* cups, with each differential segment having a length of s and the detector’s total weight being G. The weight borne by a single cup $\frac{G}{n}$ can be analyzed by considering a small segment of the cup in the figure, denoted as G_x_:

(2) $$G_{x}=\frac{G\times d\theta }{2n\pi }$$

For the analysis of the normal force on the contact surface:

(3) $$P^{\prime }\times s\times D\times \frac{d\theta }{2}=\frac{G\sin\theta }{2n\pi }d\theta$$

The compressive force acting on this part is:

(4) $$P^{\prime }=\frac{G\sin\theta }{n\pi sD}$$

In this study, the MFL detector has four plates and cups. When operating in a horizontal pipeline, the compressive force per unit area due to gravity is:

(5) $$P^{\prime }=\frac{G\sin\theta }{4\pi xD}$$

The sliding friction force generated by gravity in this part of the detector during operation is:

(6) $$F_{f1}^{\prime }=\mu \frac{G\sin\theta }{4}$$

From the above equations, it is evident that the compressive force due to gravity on the MFL detector’s plates and cups varies in different directions, reaching its maximum at the bottom of the driving cups [6].

### 2.3. Analysis of Resistance at the Cup End of the MFL Detector

**Figure 4:**
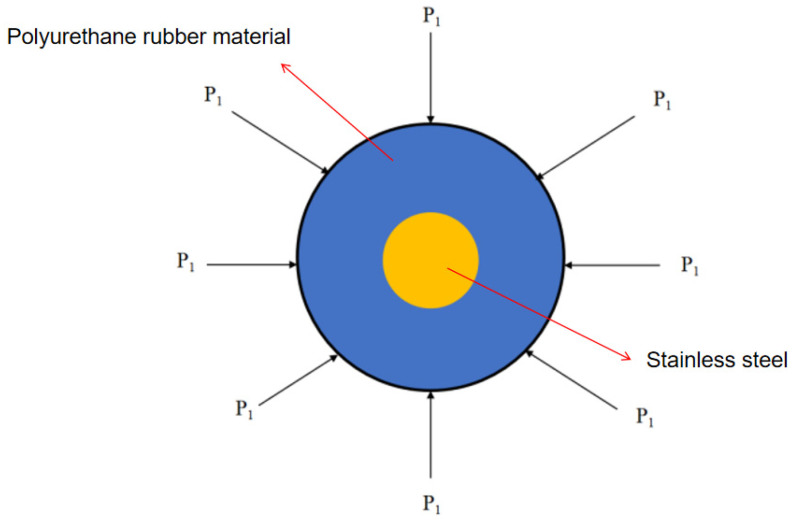
Force analysis at the cup end.

The analysis of the cup end is presented below, and the force analysis is illustrated in Figure 4.

Assuming uniform deformation, the radial deformation of the cup under external pressure is:

(7) $$\varepsilon =-\frac{D}{2E}[\frac{\frac{D^{2}}{4}+{\left(\frac{D}{2}-b\right)}^{2}}{\frac{D^{2}}{4}-{\left(\frac{D}{2}-b\right)}^{2}}-\lambda ]P_{1}$$

The pressure received by the cylindrical lip of the cup is:

(8) $$P_{1}=\frac{2(-Db+b^{2})\varepsilon E}{[\frac{D^{2}}{2}+(b^{2}-Db)(1+\lambda )]D}$$

**Figure 5:**
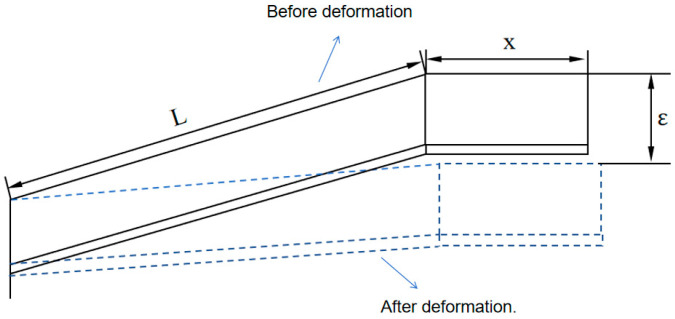
Diagram of cup end as a cantilever beam.

The cantilever beam is shown in Figure 5. With an interference fit ε, considering the bending deformation effect, the cup end can be treated as a cantilever beam with force F applied at one end. The deflection equation for the beam is:

(9) $$w=\iint \frac{M(x)}{EI}dxdx$$

where M(x)=F(l−x),F=$P_{2}\times x\times \frac{D}{2}\times d\theta$ is the concentrated load at the free end and I is the moment of inertia:

(10) $$I=\frac{\frac{D}{2}\times d\theta \times b^{3}}{12}\times \frac{D\times b^{3}}{24}d\theta$$

Substituting into the equation:

(11) $$w=\frac{Fl^{3}}{3EI}=\frac{4P_{2}xl^{3}}{Eb^{3}}$$

The total deformation of the cup due to compression is:

(12) $$\varepsilon =\frac{4P_{2}xl^{3}}{Eb^{3}}$$

The pressure due to the deformation of the cup is:

(13) $$P_{2}=\frac{\varepsilon Eb^{3}}{4xl^{3}}$$

The total pressure on the MFL detector’s cup due to the interference fit is:

(14) $${P=P}_{1}+P_{2}=\frac{2(-Db+b^{2})\varepsilon E}{[\frac{D^{2}}{2}+(b^{2}-Db)(1+\lambda )]D}+\frac{\varepsilon Eb^{3}}{4a{\left(L+\frac{x}{2}\right)}^{3}}$$

The compressive force is:

(15) $$F_{N}=P\times S$$

The formula for the resistance F experienced by the cup in a horizontal pipeline is as follows:

(16) $$F_{f}=\mu F_{N}=\left\{\frac{2(-Db+b^{2})\varepsilon E}{[\frac{D^{2}}{2}+(b^{2}-Db)(1+\lambda )]D}+\frac{\varepsilon Eb^{3}}{4a{\left(L+\frac{a}{2}\right)}^{3}}\right\}\mu n\pi Dx$$

In the formula, D is the diameter of the pipeline in mm; ε is the interference fit of the leather bowl in mm; b is the thickness of the leather bowl in mm; E is the elastic modulus of the leather bowl material in Pa; λ is the Poisson’s ratio of the leather bowl material; L is the length of the lip of the leather bowl in mm; x is the contact length of a single leather bowl with the pipeline in mm; μ is the coefficient of friction; and n is the number of leather bowls.

The total frictional force during the movement of the MFL detector is:

(17) $$F_{f}=\mu nP\pi Da=\left\{\frac{2(-Db+b^{2})\varepsilon E}{[\frac{D^{2}}{2}+(b^{2}-Db)(1+\lambda )]D}+\frac{\varepsilon Eb^{3}}{4a{\left(L+\frac{a}{2}\right)}^{3}}\right\}\mu n\pi Da$$

**Figure 6:**
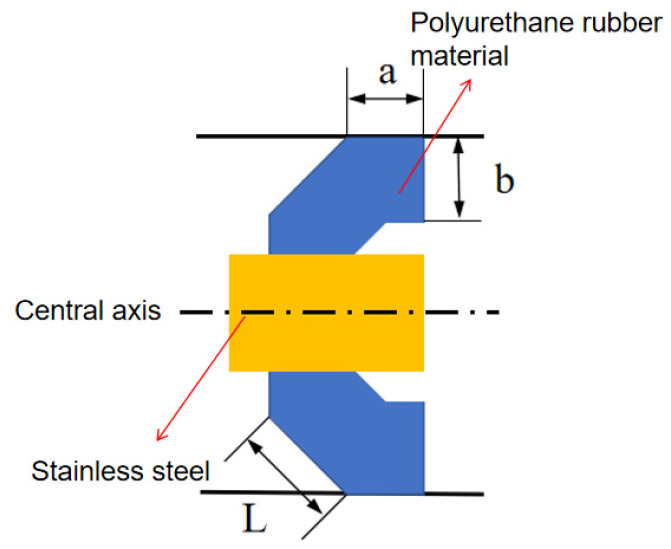
Cup Structure Diagram.

During the operation of the MFL detector, the friction coefficient is assumed to be constant. According to the mechanical design manual, the elastic modulus E is taken as 1.07 G Pa, λ = 0.4 and the friction coefficient for the steel pipe is μ = 0.07. Using the established mathematical model, the frictional force at the cup end of the MFL detector’s driving section can be calculated. For example, using an experimental platform with a pipeline outer diameter of 273 mm, the cup undergoes forced deformation due to the interference fit during operation, resulting in radial compression and an increased contact area with the pipeline wall. The structure and parameter inside the pipeline are presented inis as Figure 6 and Table 2: 

In practical engineering, the interference fit is generally used to represent the relative size between the MFL detector and the pipeline, expressed as:

(18) $$\delta =\frac{D_{j}-D}{D}$$

where δ is the interference fit of the driving section of the MFL detector, $D_{j}$ is the diameter of the driving section, and D is the inner diameter of the pipeline.

**Figure 7:**
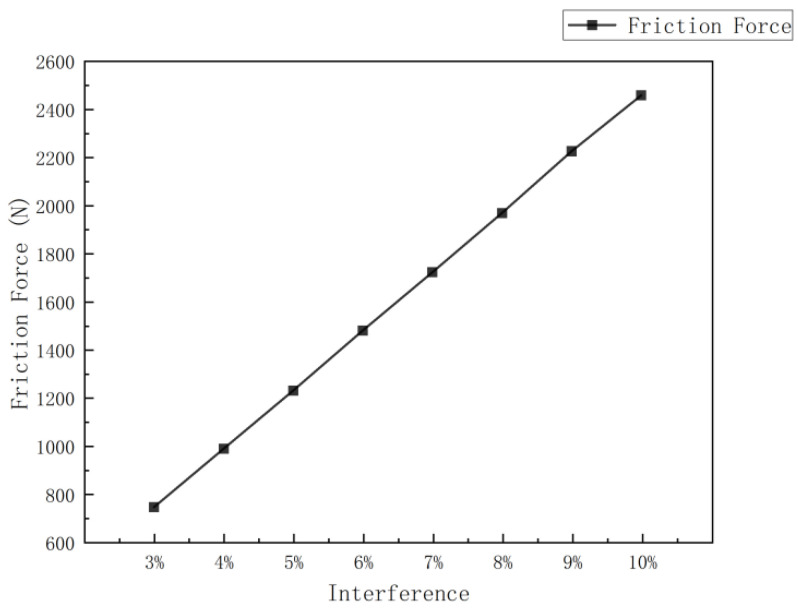
Graph of cup frictional force vs. interference fit.

Using the above formula, and substituting the parameters of the cup, the relationship between the frictional force of the cup and the interference fit is obtained as seen in Figure 7: 

The graph shows that, with all other conditions being constant, the frictional force experienced by the cup increases with the interference fit. The rate of increase becomes more significant as the interference fit grows. This is because the diameter of the MFL detector exceeds the inner diameter of the steel pipeline, causing forced deformation upon entry. The resulting normal pressure from the pipeline wall increases with greater interference, leading to increased frictional resistance during movement.

### 2.4. Analysis of Resistance at the Straight Plate End of the MFL Detector

**Figure 8:**
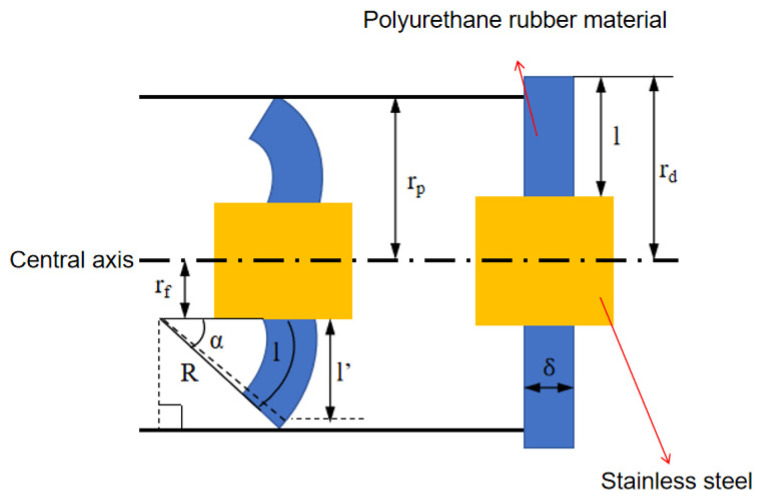
Structure of the straight Ppate.

The analysis of the straight plate end is presented in Figure 8: 

Assuming the radial length of the straight plate is l, and ignoring radial compression, the projected radial length l′ can be calculated as follows:

(19) $$l^{\prime }=Rsin\alpha =\frac{r_{d}-r_{f}}{\alpha }=r_{p}-r_{f}-\frac{\delta }{2}sin\alpha$$

where $l^{\prime }$ is the radial projection length of the straight plate centerline (m); $r_{d}$ is the radius of the straight plate end (m); $r_{p}$ is the inner radius of the pipeline (m); $r_{f}$ is the radius of the straight plate end (m); δ is the thickness of the straight plate end (m); and α is the bending angle of the straight plate end (°).

The formulas for calculating the contact length △x and contact area S between the scraper sealing plate and the pipeline wall are:

(20) $$∆x=\frac{l^{\prime }}{tan\alpha }-\sqrt{{\left(R+\frac{\delta }{2}\right)}^{2}+l^{\prime 2}}$$

(21) $$S=2\pi r_{p}∆x$$

Selecting an infinitesimal element with a central angle θ along the radial section of the straight plate end, the moment M_f_ due to frictional force F_f_ at point A is calculated as:

(22) $$M_{f}=F_{f}(r_{p}-r_{f})r_{p}d\theta$$

where $M_{f}$ is the moment due to friction, N·m, and $F_{f}$ is the frictional force per unit length of the straight plate, N/m.

In a natural gas pipeline, the moment due to the normal pressure of the pipeline wall at point A, according to Coulomb’s law, is:

(23) $$M_{w}=\frac{F_{f}}{\mu }[R(1-\cos\alpha )-\frac{\theta }{2}\cos\alpha ]r_{p}d\theta$$

where μ is the friction coefficient of the gas pipeline and $M_{w}$ is the moment due to the normal pressure of the pipeline wall, N·m.

The moment at point A due to compressive bending stresses $\sigma_{c}$ and tensile bending stresses $\sigma_{t}$ is calculated as follows:

(24) $$M_{c}=M_{t}=\frac{EI}{R}=\frac{r_{f}\delta^{3}}{24R}Ed\theta$$

where E is the elastic modulus of the straight plate (Pa); $M_{c}$ is the moment due to compressive bending stress (N·m); $M_{t}$ is the moment due to tensile bending stress; and I is the moment of inertia of the straight plate around point A, calculated as follows:

(25) $$I=\int_{0}^{\delta /2}y^{2}dyr_{f}d\theta =\frac{1}{24}r_{f}\delta^{3}d\theta$$

The moment at point A due to circumferential stress caused by radial compression in the straight plate within the pipeline, constrained by the interference fit, is:

(26) $$M_{\theta }=E\frac{R\delta d\theta }{1-\mu^{2}}\int_{0}^{\alpha }\frac{\beta -\sin\beta }{\beta +\frac{r_{f}}{R}}\left(1-\cos\beta \right)d\beta$$

where μ is the Poisson’s ratio.

During the calculation, assuming the MFL detector is in a steady state with balanced forces, the sum of internal and external moments equals zero. The total moment balance equation is:

(27) $$\sum M=(M_{f}+M_{w})-(M_{c}+M_{t}+M_{\theta })=0$$

The frictional force $F_{f}$ on the infinitesimal element dθ of the straight plate end can be derived from the above equation:

(28) $$F_{f}=\frac{M_{c}+M_{t}+M_{\theta }}{(r_{p}-r_{f})r_{p}+\frac{1}{\mu }\left[R\left(1-\cos\alpha \right)\right]r_{p}}$$

Integrating this over the circumference yields:

(29) $$F_{f}=\int_{0}^{2\pi }\frac{M_{c}+M_{t}+M_{\theta }}{(r_{p}-r_{f})r_{p}+\frac{1}{\mu }\left[R\left(1-\cos\alpha \right)\right]r_{p}}d\theta$$

In an example, with a natural gas pipeline outer diameter of 273 mm, wall thicknesses of 6 mm, 7 mm, and 10 mm, a straight plate end diameter of 273 mm, a thickness of 20 mm, an elastic modulus of 20 MPa, and a Poisson’s ratio of 0.5, the frictional forces can be calculated as Table 3: 

Using MATLAB R2024a software, the results obtained are shown in the table. With a constant thickness and material properties of the straight plate, the frictional force at the straight plate of the MFL detector is positively correlated with the pipeline wall thickness. During operation, the straight plate must bend to some extent to move normally within the pipeline. The bending angle of the straight plate increases with the interference fit, further increasing the frictional resistance at the straight plate stage.

It has been measured that the weight of the MFL detector is approximately 50 kg. When simulating the movement of the internal detector within the steel pipe, through analysis, it is known that the influence of the vertical component of the gravitational force on the frictional force during the movement of the internal detector is less than 5% (that is, the influence caused by gravity is significantly smaller than the contact stress). Therefore, in this case, it is reasonable to neglect the influence of gravity on the movement of the detector and adopt the gravity-free approximation to simplify the model, facilitating analysis and simulation. Meanwhile, when the detector is moving at a high speed, for example, when the speed is greater than 5 m/s, according to Newton’s second law, if the gravitational force compared with other forces (such as the force generated by the driving pressure difference and the frictional force) satisfies a certain condition (the specific condition can be further detailed and added here if needed), it can be approximately considered that the influence of gravity on the motion state of the detector is negligible, and the gravity-free approximation can also be adopted at this time. In addition, when analyzing the interaction between certain components inside the detector, if the forces acting on these components in the vertical direction are mainly dominated by other factors, the influence of gravity can be ignored. For instance, the magnets inside the detector are evenly distributed, generating a relatively balanced magnetic attraction force on the pipeline wall. Due to the strong magnetic attraction, most of the gravitational components in the vertical direction are offset. Thus, the influence of gravity can be neglected in the analysis of the frictional resistance of the internal detector.

## 3. Numerical Simulation

Based on the structure and force analysis of the MFL detector, a corresponding finite element model is established using ABAQUS software. Ignoring the effects of gravity, the frictional deformation at the cup end and straight plate end during operation in a gas pipeline is simulated, yielding the frictional resistance between the detector’s driving section and the steel pipeline wall. The parameters of the driving section are shown in Table 4.

The parameters of the MFL detector’s driving section under actual operating conditions are shown in the table. Using the previously established mathematical model, the frictional resistance of the driving section is calculated as Table 5: 

Based on the structure and stress analysis of the in-line inspection tool, an equivalent finite element model was created using the ABAQUS software. Ignoring gravitational effects, the model simulates and analyzes the frictional deformation between the cup end and the flat end of the inspection tool as it operates within a gas pipeline, outputting the frictional resistance between the dynamic seal of the tool and the steel pipe wall.

Currently, the dynamic cups and flat ends of the inspection tool primarily use polyurethane rubber due to its high elasticity, hardness, tear resistance, and abrasion resistance. Given its hyperelastic and incompressible properties, the simulation results are highly influenced by the choice of constitutive model and material parameters. To further investigate the impact of linear and nonlinear constitutive models on the mechanical state, a nonlinear material model is employed in this study. The Shore hardness of the polyurethane rubber is obtained via a durometer, and constitutive parameters are calculated through empirical formulas to achieve more accurate material settings.

As a typical hyperelastic material, polyurethane rubber can be represented by various constitutive models [8]. Two common models include the molecular chain network model based on thermodynamic statistical methods and the phenomenological model based on continuum mechanics [9]. The former, which encompasses Gaussian and non-Gaussian statistical models, posits that the molecular structure of hyperelastic materials is disordered when not subjected to external forces, with entropy decreasing as the tensile force increases. This model reflects the polymer physics of the material, allowing material parameters to have physical significance. However, it requires detailed material property data, limiting its practical application. The second type, based on the phenomenological theory of continuum mechanics, includes the Mooney–Rivlin, Neo-Hookean, and Ogden models. This approach uses basic material tests such as uniaxial and biaxial stretching to define the stress–strain relationship of hyperelastic materials, and the strain energy function is derived through data fitting.

In this study, the phenomenological theory of continuum mechanics is employed based on the following assumptions: (1) isotropy—polyurethane rubber possesses identical properties in regard to strength, stiffness, and elongation in all directions; (2) incompressibility—the material’s density remains unchanged during deformation. The main forms include polynomial models, the Mooney–Rivlin model, the Neo-Hookean model, and the Yeoh model.

As a commonly used form of strain energy potential function, the polynomial model can be expressed as:

(30) $$W=f(I_{1}-3,I_{2}-3)+g(J-1)$$

Expanding this equation in a Taylor series yields:

(31) $$W=\sum_{i+j=1}^{N}C_{ij}{\left(I_{1}-3\right)}^{i}{\left(I_{2}-3\right)}^{j}+\sum_{i=1}^{N}\frac{1}{D_{i}}{\left(J-1\right)}^{2i}$$

where N represents the model order, W the strain energy per unit reference volume, C_ij_ the material shear performance parameter, $D_{i}$ the compression performance parameter, $I_{1}$ and $I_{2}$ the first and second strain invariants, and J the elastic volume ratio. If the material is considered fully incompressible, all D_i_ values default to zero and the second part of Equation (31) is omitted [10].

The Mooney–Rivlin model is a simplified version of the polynomial model. It is derived through shear tests on rubber materials under less than 100% deformation following these assumptions: (1) rubber is isotropic and incompressible; (2) under shear deformation, it obeys Hooke’s law, where strain and stress are linearly related. Mooney first proposed a model based on $I_{1},I_{2}$, with the strain energy function expressed as:

(32) $$W=C_{10}(I_{1}-3)+C_{01}(I_{2}-3)$$

Rivlin extended this by replacing the terms with higher-order expressions with the strain energy function as:

(33) $$W=\sum_{i,j=0}^{\infty }C_{ij}{\left(I_{1}-3\right)}^{i}{\left(I_{2}-3\right)}^{j}$$

where $C_{10},C_{01}$, $C_{ij}$ are material shear performance parameters and $C_{00}$ = 0.

In the Neo-Hookean model, $C_{01}$ = 0, and its expression is:

(34) $$W=C_{10}(I_{1}-3)$$

The Neo-Hookean model is the simplest form of strain energy function and an extension of Hooke’s law that is suitable for small to medium deformations in rubber but not for large deformations. This model, derived through shear tests, is widely applicable in shear mechanics. Due to its single material parameter, results are relatively stable, especially when fitting the stress–strain curve under a single loading condition.

The Yeoh model is expressed as:

(35) $$W=\sum_{i=1}^{3}C_{i0}{\left(I_{1}-3\right)}^{i}$$

The Yeoh model overcomes the drawback that the Mooney—Rivlin model has in regard to poor performance in describing the hardening ability of rubber under large-deformation conditions. $\frac{\partial W}{\partial I_{1}}$ is much larger than $\frac{\partial W}{\partial I_{2}}$. As the strain variable increases, $\frac{\partial W}{\partial I_{2}}$ gradually decreases and tends towards zero. It can describe the strain conditions of rubber materials under various working conditions; however, its accuracy is poor in terms of fitting biaxial tensile tests.

Due to each rubber material constitutive model’s unique advantages, disadvantages, and applicable range, this study employs the Mooney–Rivlin model for analyzing polyurethane cups, given the model’s suitability for small-to-medium deformations, which aligns with the limited interference fit of the polyurethane cup.

In this study, the flat and cup ends of the dynamic segment use polyurethane rubber material, and their response to force during operation relates to their hardness. Using empirical formulas, the rubber hardness was converted into the Mooney–Rivlin elastic parameters $C_{10}$ and $C_{01}$, significantly reducing testing costs [11,12,13].

For small deformations, the relationship between polyurethane rubber’s elastic modulus and shear modulus satisfies the following equation:

(36) $$G=\frac{E_{0}}{2(1+\mu )}$$

where is Poisson’s ratio of the material.

Assuming an incompressible property for rubber, μ = 0.5, thus $E_{0}$ = 3G, and the relationship between shear modulus, elastic modulus, and $C_{10},C_{01}$ is shown below:

(37) $$G=\frac{E_{0}}{3}=2(C_{10}+C_{01})$$

(38) $$E_{0}=6(C_{10}+C_{01})$$

Based on IRHD hardness H and elastic modulus $E_{0}\;$experimental data for rubber materials, the fitting provides:

(39) $$lgE_{0}=0.0198H-0.5432$$

The above equation shows that IRHD hardness depends on Rivlin parameters $C_{10}$ and $C_{01}$.

Shore Hardness, a widely recognized indicator of material deformation under compression or puncture resistance, measures how far a steel indenter penetrates the material’s surface when fully engaged. The Shore hardness, represented by L, inversely correlates with hardness: a higher L indicates lower hardness and vice versa. Shore hardness type HA represents softer rubber, while HD represents harder rubber or plastics. The polyurethane rubber used in this study applies to HA-type Shore hardness.

**Figure 9:**
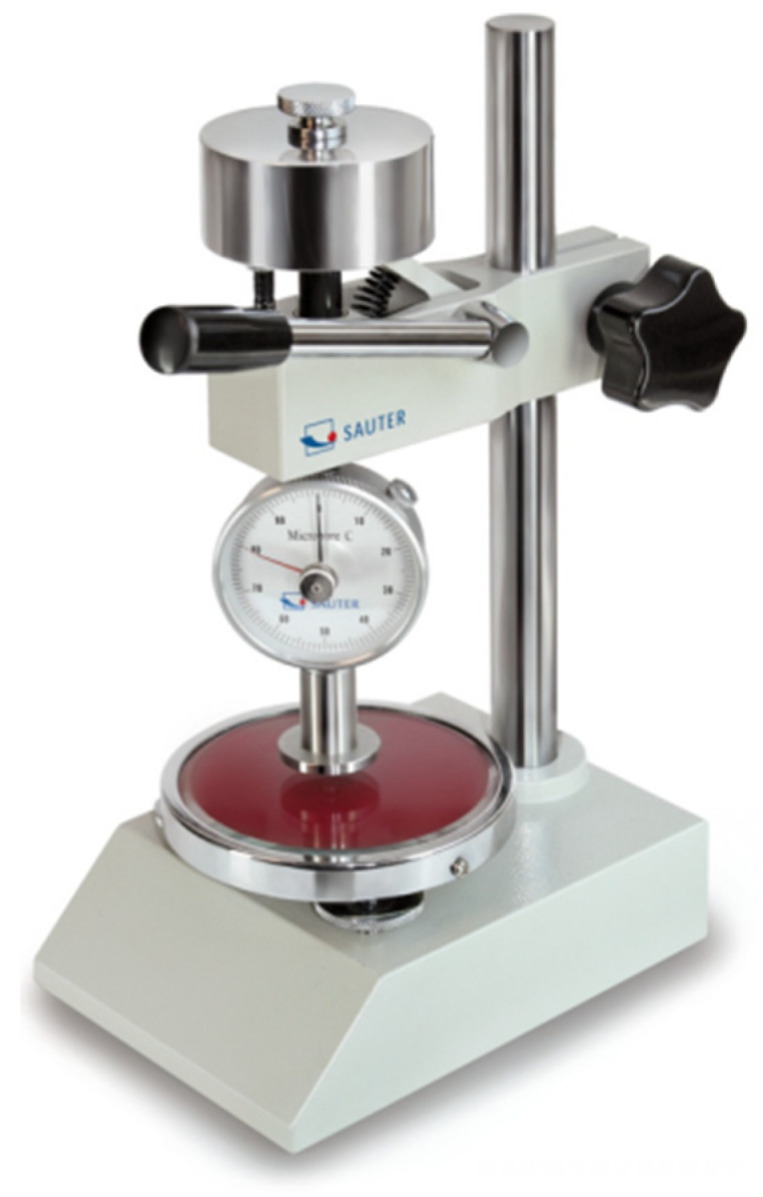
Shore Scleroscope.

Tester experimental procedures and standards for the Shore hardness tester (as shown in Figure 9) followed GB531, and the results are listed in Table 6.

Calculating the average hardness value for the sealing element provides the Rivlin coefficients for polyurethane rubber at the flat and cup ends, with C_10_ being set to 1.423 and C_01_ being set to 0.356.

The main body of the dynamic segment and pipeline materials, composed of steel, undergo minimal deformation during operation. Thus, a linear elastic constitutive model was selected with an elastic modulus of E = 200 MPa and a Poisson’s ratio of μ = 0.3.

**Figure 10:**
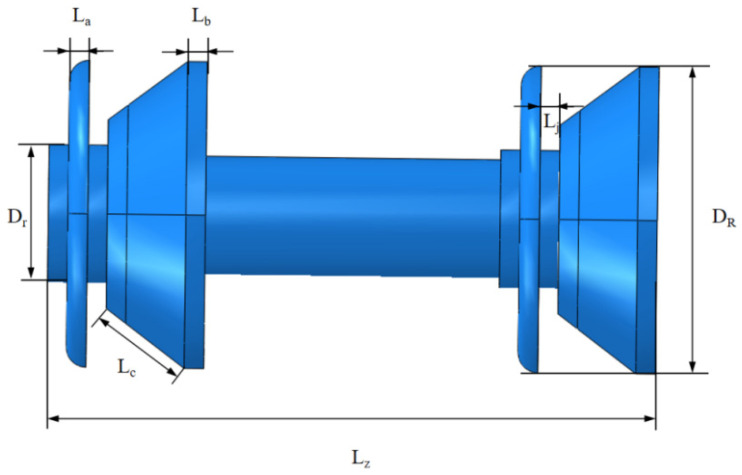
Geometric model of the driving section.

The driving section structure selected for this study is a common mandrel type, as shown in Figure 10. The main components include cups, straight plates, mandrels, and clamping plates. During assembly, the straight plates and cups are sequentially fitted onto the mandrel, with clamping plates being installed on both sides for fixation.

This study analyzes the driving section under different pipeline wall thickness conditions, as shown in Table 7.

**Figure 11:**
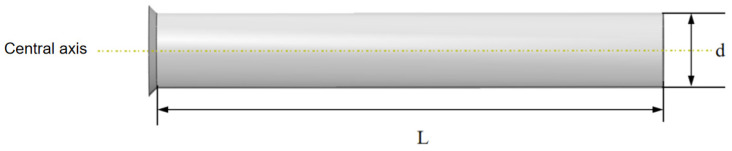
Geometric model of the pipeline.

In this study, the parameters of the driving section remain constant under different conditions, with the variation in interference fit being primarily influenced by changes in the inner diameter of the steel pipeline. The geometric model of the pipeline is shown in Figure 11.

**Figure 12:**
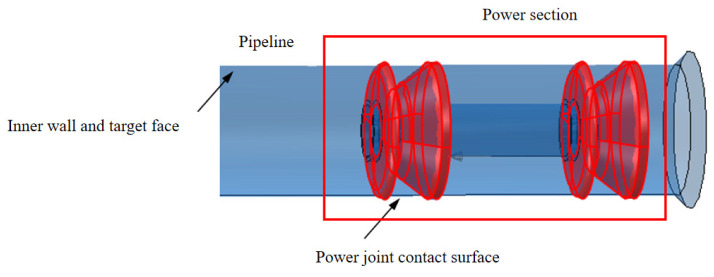
Contact surface and target surface diagram.

**Figure 13:**
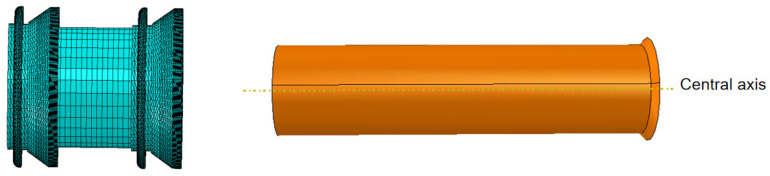
Mesh division diagram of the driving section and pipeline.

The study uses ABAQUS software to establish a fluid–structure interaction model of the MFL detector’s driving section in a straight gas pipeline, as shown in Figure 12. The main body of the model is meshed using regular C3D8 elements, presenting a well-organized cubic structure. For the edge regions of the model (cup area), regular C3D4 elements are used to accommodate the complex boundary geometry. As shown in Figure 13, the straight plate and cup ends of the driving section are fixed with clamping plates and no displacement occurs between components, hence binding contact is used. Face-to-face contact is selected between the straight plates, cups, and the pipeline using the penalty function algorithm. The Coulomb friction coefficient between the straight plates, cups, and the pipeline inner wall is set to 0.5 [14].

In the model, the gas pipeline and the main shaft of the MFL detector are made of steel, which, compared to the polyurethane rubber material of the straight plate and cup ends, undergoes negligible deformations. To further simplify calculations, the steel pipeline and the main body are coupled as rigid bodies. In this study, the steel pipeline is set with full constraint conditions, remaining stationary. The driving section of the detector is subjected to speed loading, set at 30 mm/s, and solved using the Explicit solver with a quasi-static approach.

**Figure 14:**
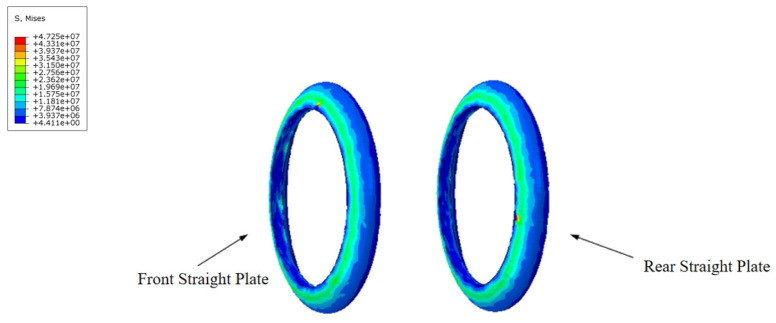
Comparison of contact stress at the front and rear straight plates of the driving section.

**Figure 15:**
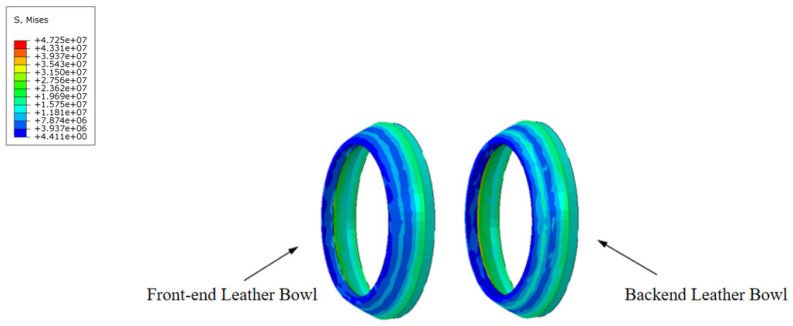
Comparison of contact stress at the front and rear cups of the driving section.

During the movement of the MFL detector’s driving section in the steel pipeline, the cup and straight plate ends closely adhere to the steel pipeline. The contact stress between them directly reflects the degree of adherence and the magnitude of the operating friction. Taking a driving section component with an interference fit of 4.6% as an example, when the driving section fully enters the steel pipeline and stabilizes, the contact stress distribution of each component is shown in Figure 14 and Figure 15. The contact stress size and distribution of the front and rear components are approximately the same. To further analyze the contact stress distribution under different interference fits, the front half of the driving section’s straight plate end and cup segment is selected for discussion.

**Figure 16:**
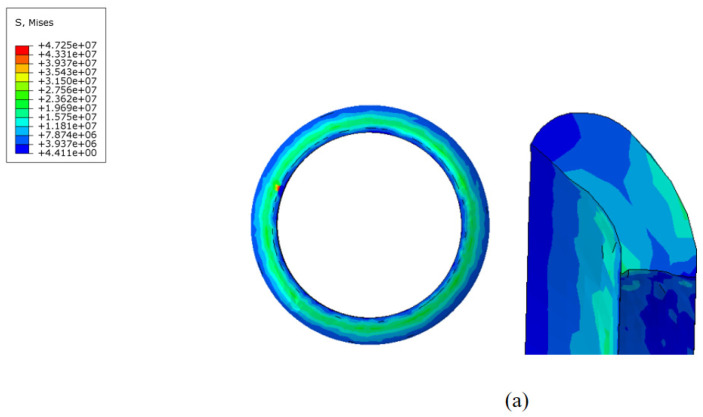

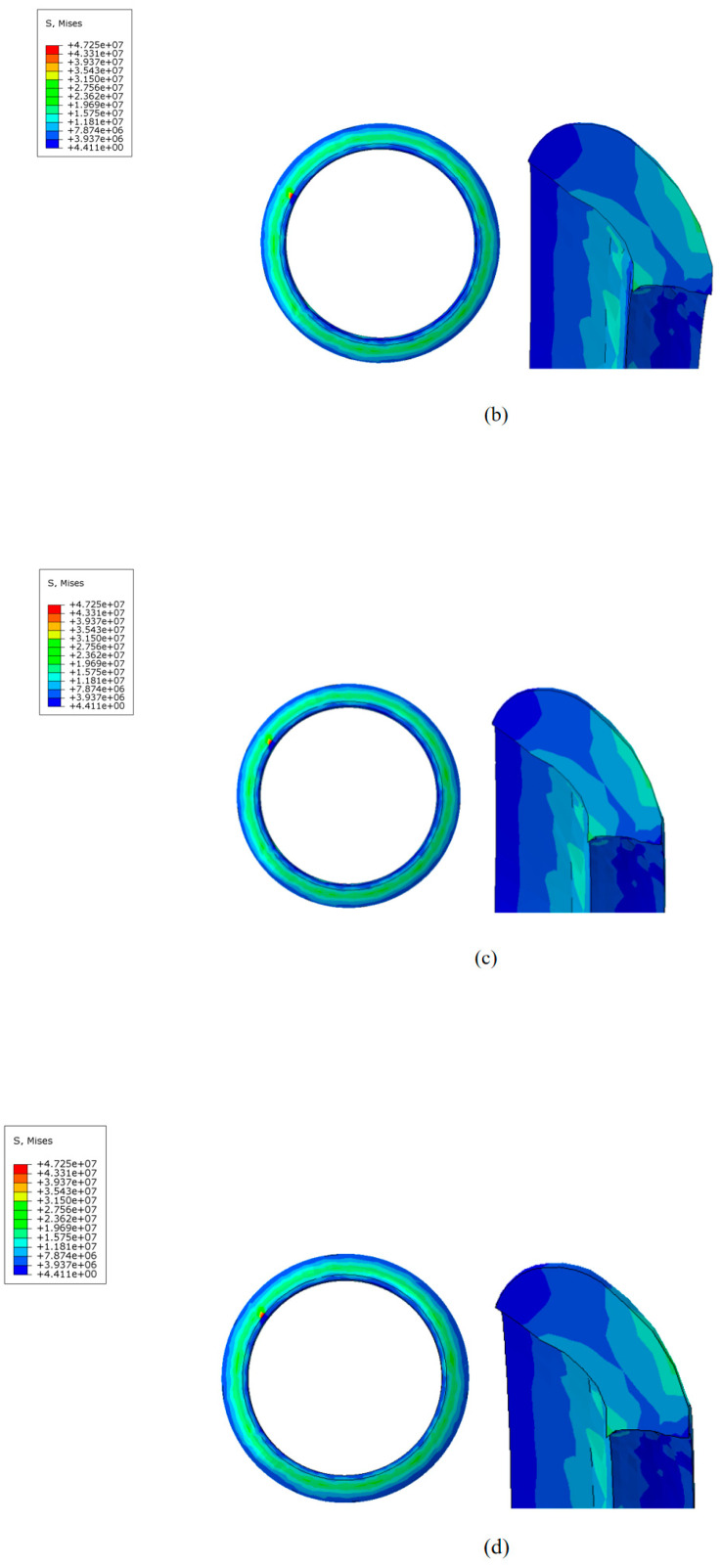

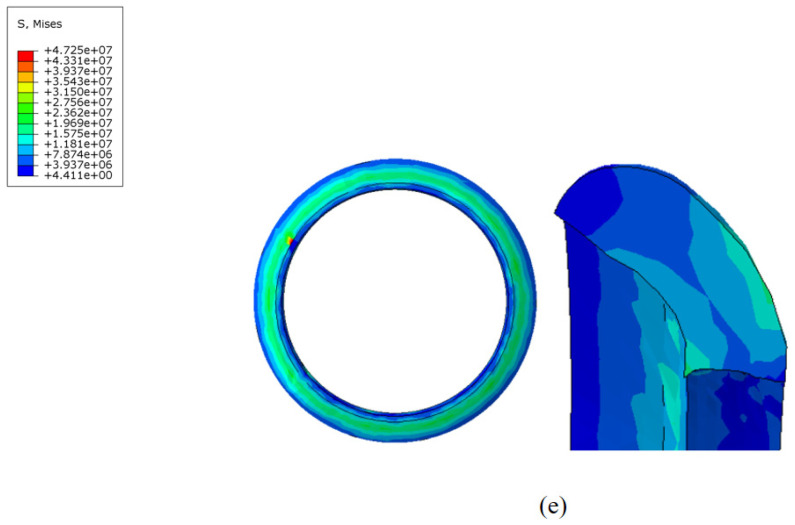
The contact stress of the flat end as a function of interference fit: (**a**) flat end with 6 mm wall thickness; (**b**) flat end with 7 mm wall thickness; (**c**) flat end with 8 mm wall thickness; (**d**) flat end with 9 mm wall thickness; (**e**) flat end with 10 mm wall thickness.

The contact stress and distribution at the straight plate end under different interference fits are shown in Figure 16. When the MFL detector operates within the pipeline, the contact force distribution at the straight plate end under various interference conditions is similar. Due to the interference fit, the straight plate end is compressed by the pipeline wall, with maximum contact stress occurring at the outer edge of the straight plate end, decreasing from the outside in. Comparing different conditions reveals that, as the interference fit increases, the degree of bending deformation at the straight plate end increases, further raising the contact stress.

**Figure 17:**
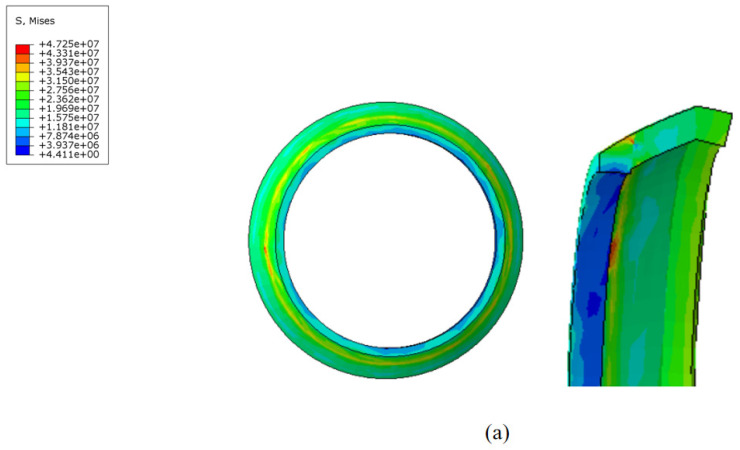

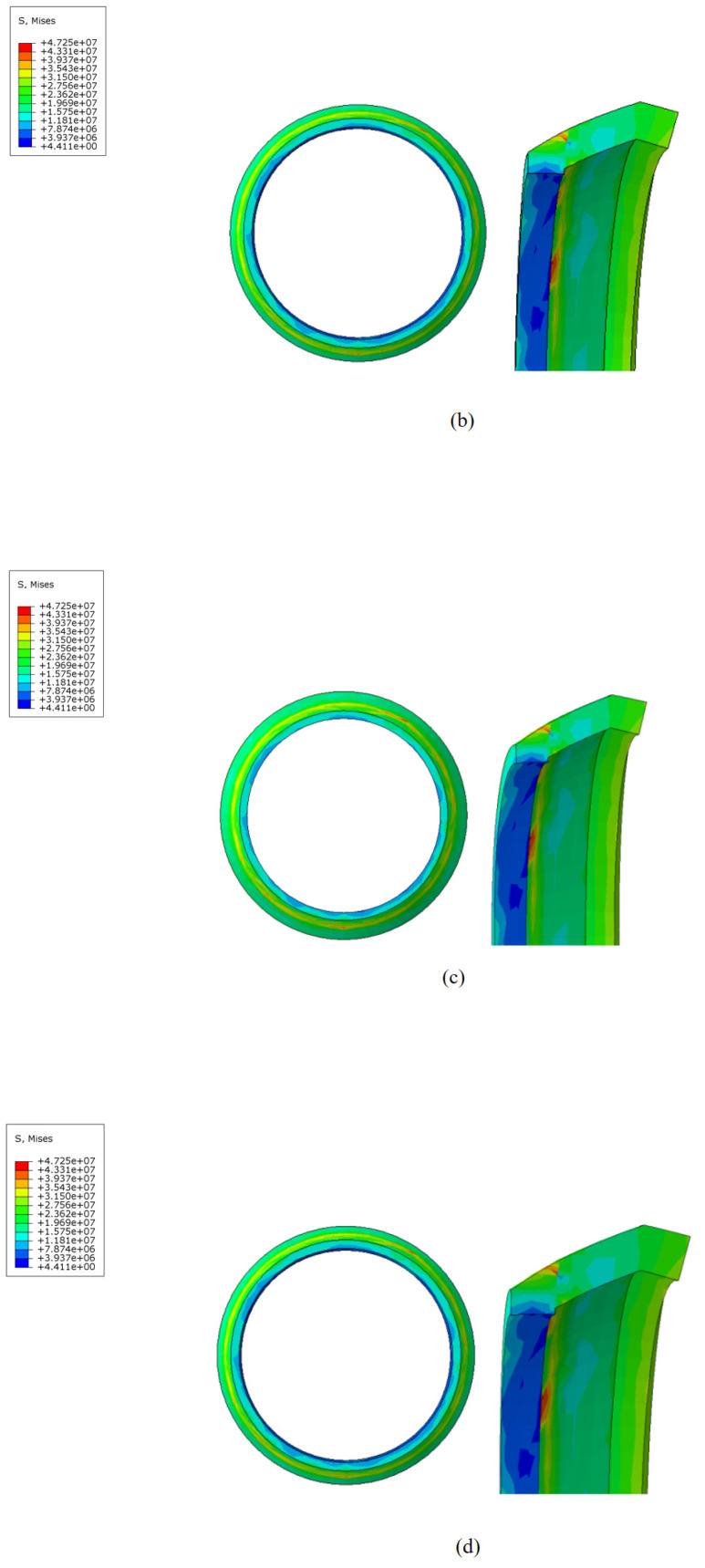

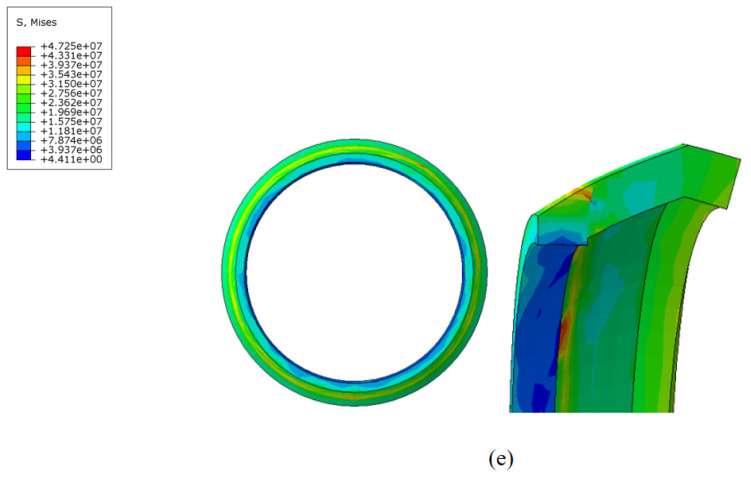
Contact stress at the end of the leather bowl versus interference fit: (**a**) cup with 6 mm wall thickness; (**b**) cup with 7 mm wall thickness;(**c**) cup with 8 mm wall Thickness; (**d**) cup with 9 mm wall thickness; (**e**) cup with 10 mm wall thickness.

**Figure 18:**
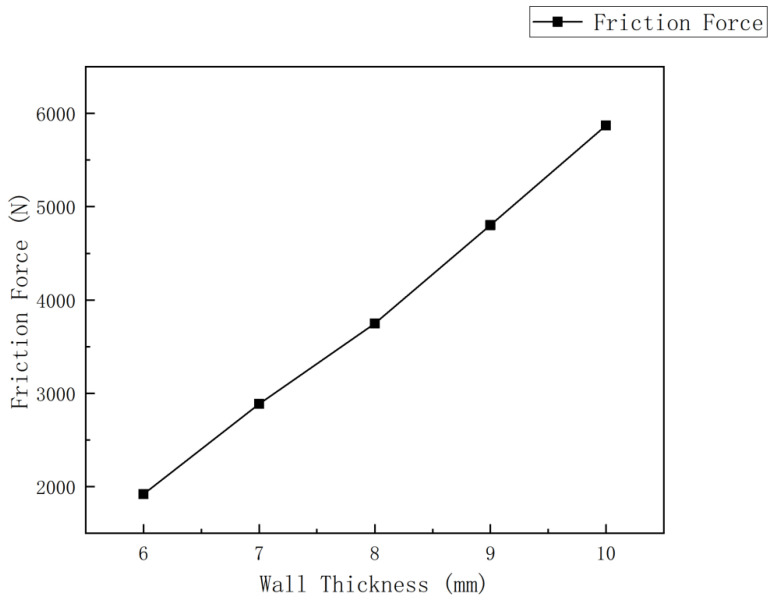
Variation in frictional resistance in the driving section with the wall thickness of a 273 mm pipeline.

The contact stress at the cup end under different interference fits is shown in Figure 17. Under various interference conditions, the contact stress distribution at the cup end of the MFL detector’s driving section is relatively similar. Due to the interference fit, the cup end does not remain tightly attached to the steel pipeline wall but is compressed, with the front end of the cup being pressed down and tightly contacting the steel pipeline wall at the fold of the cup end. This differs somewhat from the theoretical conditions considered earlier. When the interference fit is small, the maximum contact stress at the cup end is at the end of the contact position with the steel pipeline. As the interference fit increases, the maximum contact stress and position move towards the end of the cup, and, as the interference fit increases, the contact position and maximum contact stress at the cup end move further down. The variation in frictional resistance in the driving section with the wall thickness of a 273 mm pipeline is shown in Figure 18.

## 4. Experimental Study of Natural Gas In-Line Inspection

During actual operation, the MFL detector is driven by the pressure differential of the medium in front of and behind the cups. As it moves forward, it uses the attached detection device to locate pipeline defects, overcoming the frictional resistance with the pipeline wall. To measure the magnitude of the frictional resistance and verify the proposed frictional resistance model, an experimental platform was established. By comparing the theoretical model with the actual measured frictional forces, the model is refined to improve its accuracy.

### 4.1. Experimental System Design

An experimental platform for pulling the MFL detector was established, and methods for testing the starting force and running force were proposed. In this experiment, a winch was used to pull the detector at a constant speed, simulating the uniform motion driven by gas pressure and frictional resistance during the inspection process, thus allowing for the measurement of frictional forces. The experimental platform was constructed at the test site for pulling the MFL detector and experimental research was conducted. The MFL detector was used as the pulling object to study how its parameters affect frictional forces during movement. The detector was designed to pass through gas steel pipes of varying wall thicknesses to explore how the interference fit affects frictional resistance, and further experimental research and analyses were conducted by altering related parameters. This experiment used a mechanical one-dimensional motion platform to fix the detector’s direction of movement, allowing for horizontal movement of the inspection device and dynamic detection of the maximum static friction and sliding friction resistance of the device. During the pulling process, both whole-machine and disassembled pulling methods were used to record the frictional forces of the entire device and its individual sections, facilitating separate analyses and achieving precise quantification of the frictional force mathematical models for each component.

#### Composition of the Pulling Experimental Platform

**Figure 19:**
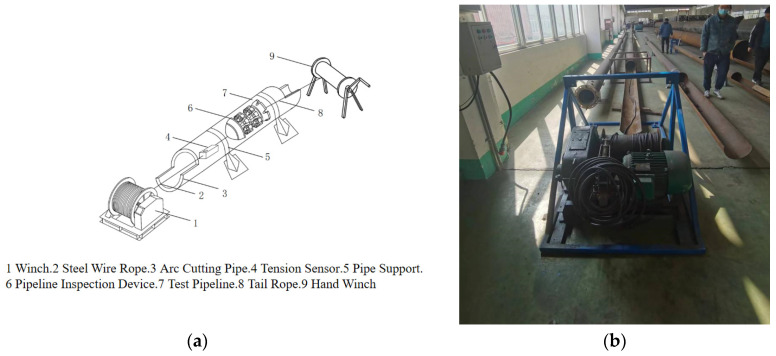
(**a**) Experimental device diagram; (**b**) site plan diagram.

This experimental platform was designed and constructed with reference to common pipeline specifications in urban gas networks. As shown in Figure 19, during the experiment, the MFL detector was placed inside a steel pipeline and a winch was used to pull a steel cable to move the detector horizontally at a constant speed of 0.5 m/s. During the inspection, a tension sensor was used to measure the pulling force and real-time data were recorded through a data acquisition system for a subsequent in-depth analysis. The MFL detector was pulled through steel pipes with wall thicknesses of 6 mm, 7 mm, and 10 mm, stopping once in each segment. A wireless tension sensor recorded the maximum static friction and sliding friction values of the detector under different interference fits, exploring the influencing factors and patterns of pipeline friction.

The entire experimental platform consists of key components such as the experimental pipeline, winch, steel cable, MFL detector, tension sensor, and power distribution device.

The experimental pipeline consists of seamless steel pipes made of L290N material, with an outer diameter of 273 mm and a total length of 60 m, including 20 m segments of Φ 273 mm × 6 mm, Φ 273 mm × 7 mm, Φ 273 mm × 10 mm. All experimental pipes were manufactured according to the GB/T 9711-2017 [15] PSL2 series standard for petroleum and natural gas pipelines. To facilitate the placement and removal of the MFL detector, flared ends were welded to the pipeline exits and both ends of the pipeline were symmetrically cut into 90° arcs, with each cut section being 2 m long.

**Figure 20:**
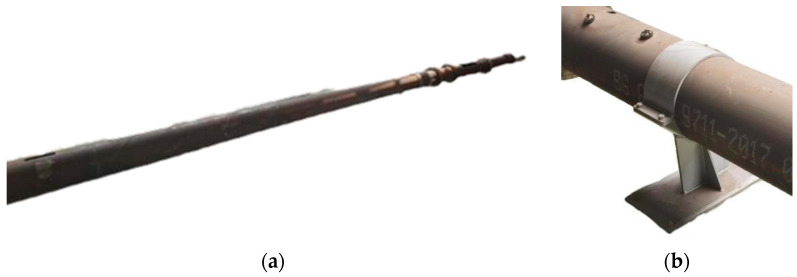
(**a**) Experimental pipeline; (**b**) pipeline support diagram.

The frictional resistance experienced by the MFL detector during the pulling process is substantial. To prevent the pipeline from being pulled off during the experiment, pipeline supports were installed. The supports were made of stainless steel, with iron pipe sleeves, 10 cm wide, being clamped on both sides with bolts. The base dimensions were 20 × 50 cm, and they were fixed with anchor bolts at the corners with a spacing of 10 m between supports, as shown in Figure 20.

**Figure 21:**
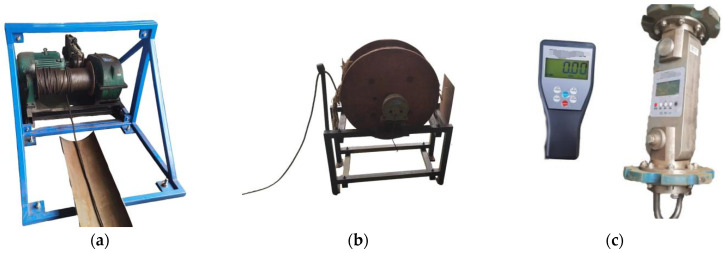
Device diagram: (**a**) traction machine and steel wire rope; (**b**) hand-operated winch and tail rope; (**c**) tensile force gauge.

The winch provides the pulling force for the entire MFL detector and facilitates the recording of pulling force values. The winch is equipped with an 11 KW three-phase asynchronous motor and a variable frequency device, allowing the detector to move uniformly through the pipeline at a speed of 0.5 m/s. An 11 mm diameter steel cable was chosen for the pulling rope, offering sufficient tensile strength to prevent breakage during pulling and ensuring safety, as shown in Figure 21a.

After each pull, the pulling rope needs to be returned to its original position in the pipeline to facilitate the next pulling experiment. To reduce the workload, a lightweight hemp tail rope was used, wound by a hand winch, with a length of 65 m, as shown in Figure 21b.

The measurement and reading of the pulling force values are achieved using a tension meter which consists of two parts: a pulling end device and a handheld device. During the pulling process, the values measured by the pulling end device can be instantly transmitted to the handheld device for reading, as shown in Figure 21c.

The various parameters of the experimental platform are shown in Table 8.

The entire experimental system generally meets testing requirements, but the following issues remain: 1. The pipeline is made of seamless steel pipe with few openings, making it difficult to accurately position the MFL detector during the pulling process. 2. The tail rope is long, making manual recovery difficult. To better address these issues, the following measures were taken: 1. Observation holes were added at the front and rear of pipelines with different wall thicknesses to facilitate positioning. 2. An electric winch was used for the tail rope-pulling device to speed up recovery and simplify operations.

### 4.2. Measurement of Frictional Force of the In-Line Inspection Tool

To quantify the frictional force of the in-line inspection tool in the pipeline, a specific pulling test scheme was designed. The test pipeline segments used in the pulling test included three specifications: Φ 273 mm × 6 mm, Φ 273 mm × 7 mm, and Φ 273 mm × 10 mm.

A total of 12 pulling tests were completed on each specification segment, including three start–stop pulls of the entire tool, three start–stop pulls of the drive section, three start–stop pulls of the inspection section, and three start–stop pulls of the storage section. The maximum static friction and sliding friction forces during smooth pulling were recorded for the entire tool and each component, with the average values being taken. The 12 pulling tests were performed at the same location for start–stop operations, with a handheld tension meter being used for real-time recording. A variable frequency winch ensured a constant pulling speed. Data sampling was conducted during the transition from static to movement, with the maximum force being taken as the maximum static friction value. After starting, the pulling force was recorded during the smooth-pulling phase, excluding extreme data points at weld locations, to determine the sliding friction of the in-line inspection tool under balanced force conditions.

### 4.3. Experimental Results

**Figure 22:**
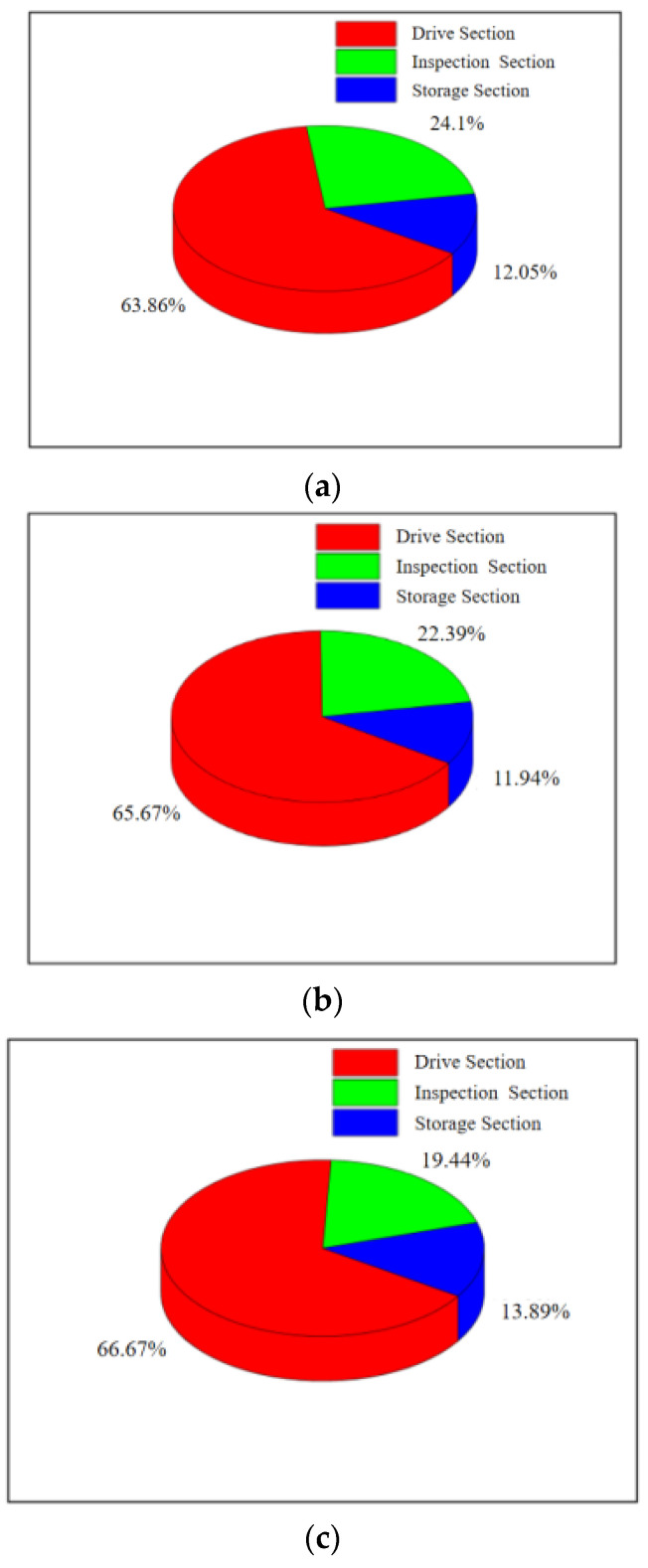

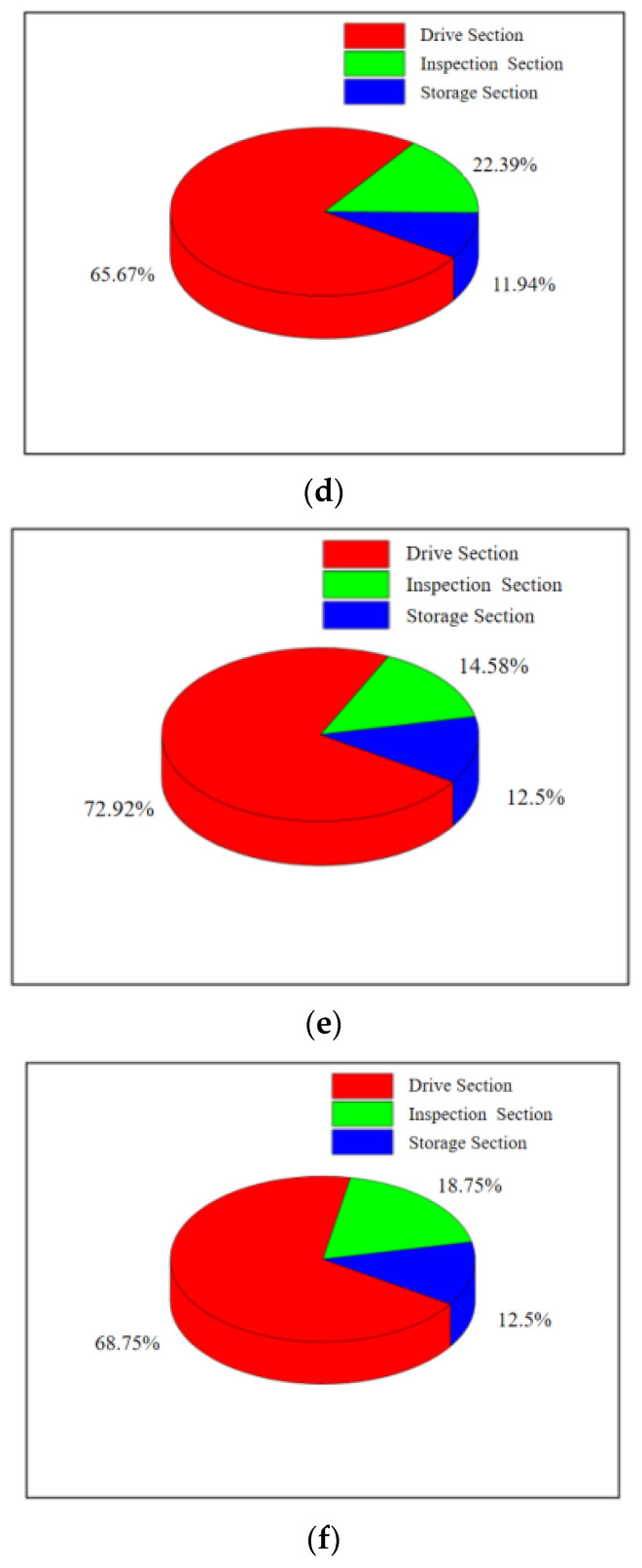
Proportion of frictional force on different parts of the in-line inspection tool: (**a**) Φ273 mm × 10 mm pipe-section-starting friction force components; (**b**) Φ273 mm×7 mm pipe-section-starting friction force components; (**c**) Φ 273 mm × 6 mm pipe-section-running friction force components; (**d**) Φ273 mm × 10 mm pipe-section-starting friction force components; (**e**) Φ273 mm × 7 mm pipe-section-running friction force components; (**f**) Φ273 mm × 6 mm pipe-section-running friction force components.

Therefore, this study considers the frictional force experienced by the in-line inspection tool during stable operation to be equivalent to the pulling force. Pulling tests were conducted using a winch in a Φ 273 mm pipeline, and the measured data were summarized and analyzed, as shown in Table 9 and Figure 22.

### 4.4. Analysis of Frictional Force of the In-Line Inspection Tool in Different Pipe Diameters

The frictional force experienced by the in-line inspection tool is caused by the contact friction between the sealing device and the pipeline. On one hand, it can directly reflect the scraping ability of the straight plate part at the front end of the tool’s drive section on the attached magazines in the pipeline; on the other hand, it can determine the magnitude of the frictional force experienced by the tool during the inspection work. The frictional forces experienced by the tool in different pipe diameters are shown in the figure. The frictional force experienced by the in-line inspection tool increases with the interference fit. During the pulling process, the starting force is greater than the running force by 18–22% under three conditions. This phenomenon is due to the fact that the polyurethane rubber cups require a greater force to induce interference deformation when forced compression occurs at the start. Additionally, the maximum static friction is greater than the sliding friction. The friction difference between static and moving processes is more pronounced in the inspection section, with a maximum margin of 67%. This is due to the magnetic attraction force between the magnetized steel brushes of the inspection section and the steel pipeline wall, which needs to be overcome at the start. Additionally, the steel brushes in the inspection section are set with interference lengths and are bent under the pressure of the pipeline wall at the start. The pressure from both these factors contributes to the starting friction of the inspection section.

## 5. Comparison and Discussion of Experimental and Simulation Results

**Figure 23:**
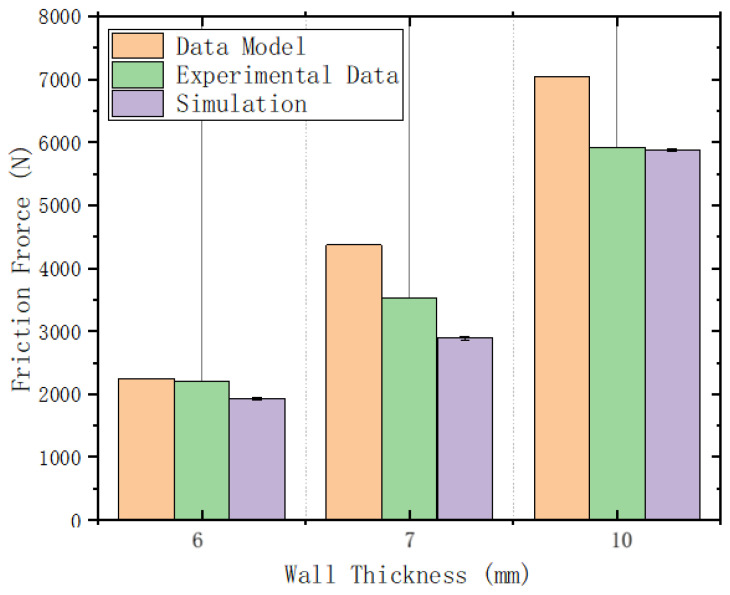
Comparison of frictional force magnitude from the mathematical model, experimental data, and simulation.

To verify the accuracy of the mathematical model and simulation, the simulation results from the previous section were compared with the experimental data in this section, as shown in Figure 23: 

As shown in the figure, the frictional force trends with pipeline wall thickness (i.e., interference fit) are similar for both the mathematical model based on engineering mechanics principles and the simulation using the Mooney–Rivlin model. However, as the pipeline wall thickness increases, the discrepancy between the mathematical model and experimental data grows, while the simulation results are closer to the experimental data. This is because, at higher interference fits, it is more challenging to capture the actual deformation data for the mathematical model, such as the bending angle of the straight plate end and the contact area of the cup end with the pipeline wall, leading to higher values in the mathematical model. In contrast, the simulation cannot replicate the uneven smoothness and manufacturing errors of the pipeline wall in a real-world environment, resulting in lower simulation values; however, it performs well at higher interference fits.

## 6. Conclusions

(1) Based on engineering mechanics theory, a force analysis was conducted on the driving section of the in-line inspection tool. Friction models for the cup end and straight plate end of the driving section were established. Different structures of cups and straight plates were analyzed, revealing the factors influencing the frictional resistance of the driving section.
(2) A fluid–structure interaction model for the driving section of the in-line inspection tool in the pipeline was established using the finite element software ABAQUS. Frictional characteristics were analyzed, especially for medium-pressure pipelines with a low driving force, providing theoretical support for developing low-friction in-line inspection tools.
(3) Pulling experiments were designed and conducted to study the frictional forces of the entire in-line inspection tool and its components during operation in the pipeline. The magnitudes of starting and running friction were analyzed, and the accuracy of the mathematical model and simulation was verified, providing solutions for selecting appropriate calculation methods under various conditions.

## Figures and Tables

**Table 1:** Parameters of the MFL Detector.

Specifications and Types	MI168	MI219	MI273	MI323	MI508	MI1016
Length (mm)	1540	1410	1680	1700	1850	1950
Weight (kg)	56	82.5	161	200	450	2600
Interference Fit	1–5%					

**Table 2:** Cup End Data.

Interference Fit	Contact Length a (mm)	Cup Thickness b (mm)	Cup Lip Length L (mm)	Number of Cups
4.6%	15	14	45	2

“a” represents: Contact Length. “b” represents: Cup Thickness. “L” represents: Cup Lip Length.

**Table 3:** Frictional Force Calculation of the Straight Plate for Different Wall Thicknesses.

Pipeline Radius	Wall Thickness	Inner Radius of Pipeline	Radius of Straight Plate	Thickness of Straight Plate	Bending Angle of Straight Plate	Frictional Resistance of Straight Plate
136.5	6	130.5	136.5	14	102.81	2929
136.5	7	129.5	136.5	14	106.12	3000
136.5	8	128.5	136.5	14	109.32	3057

**Table 4:** Geometric Parameters of the Driving Section.

Physical Parameters	Value (mm)	Physical Parameters	Value (mm)
Thickness of Straight Plate La	10	Thickness of Cup Lb	14
Thickness of Clamping Plate Lj	10	Diameter of Mandrel Dr	102
Length of Cup Lip Lc	45	Total Length of Driving Section Lz	270
Diameter of Cup DR	273		

**Table 5:** Geometric Parameters of the Driving Section.

Pipeline Segment	Φ 273 mm × 10 mm	Φ 273 mm × 7 mm	Φ 273 mm × 6 mm
Frictional Force (N)	7014	4362	2249

**Table 6:** Hardness Test Results.

Serial Number	1	2	3	4	5
Hardness Value	82	81	82	82	83

**Table 7:** Selected Interference Fit.

Condition	1	2	3	4	6
Wall Thickness	6	7	8	9	10
Interference Fit	4.6%	5.4%	6.2%	7.1%	7.9%

**Table 8:** Equipment Model Table.

Serial Number	Name	Quantity	Specifications and Technical Requirements
1	Steel Pipe	20 m	Φ 273 × 6.0
2	Steel Pipe	20 m	Φ 273 × 7.0
3	Steel Pipe	20 m	Φ 273 × 10.0
4	Arc-cut Pipe	2 sections, each 2 m	Arc Angle 90°
5	Tail Rope	65 m	Lightweight Hemp Rope with Hand Winch
6	Winch	1 unit	Rated Pulling Force 5 T, Rated Speed 0.5 m/s
7	Winch Steel Cable	65 m	Φ 11 Galvanized Steel Wire
8	Variable Frequency Controller	1 unit	Reaches Rated Speed within 5 s
9	Tension Sensor	1 unit	Maximum Range 10 T, Equipped with Hoops at Both Ends
10	Pipeline Support	6 unit	Steel Pipeline Support

**Table 9:** Pulling Data for Φ 273 mm Pipeline.

Pulling Position	Length (m)	Φ 273 mm × 10 mm			Φ 273 mm × 7 mm			Φ 273 mm × 6 mm		
		Starting Force (t)	Running Force (t)	Margin	Starting Force (t)	Running Force (t)	Margin	Starting Force (t)	Running Force (t)	Margin
Entire Tool	1.70	0.94	0.80	18%	0.62	0.51	22%	0.50	0.43	16%
Drive Section	0.43	0.63	0.59	6.8%	0.44	0.35	25.7%	0.24	0.22	9%
Inspection Section	0.40	0.20	0.12	67%	0.15	0.07	114%	0.07	0.06	16%
Storage Section	0.40	0.10	0.07	43%	0.08	0.06	33%	0.05	0.04	25%

## Data Availability

Due to the confidentiality requirements of the project responsibleunit, the data provided in this study should be provided at the request of the corresponding authorbecause the project has not yet been concluded.
